# Assessing the relation between financial performance and long-term bank loan interest rates for healthcare providers in the Netherlands: a panel data analysis

**DOI:** 10.1007/s10198-023-01629-z

**Published:** 2023-09-13

**Authors:** Erik Wackers, Rick Smit, Niek Stadhouders, Patrick Jeurissen

**Affiliations:** Radboud University Medical Center, Radboud Institute for Health Sciences, IQ Healthcare, Nijmegen, The Netherlands

**Keywords:** Healthcare costs, Financial management, Healthcare market, Healthcare reform

## Abstract

The Dutch health system is financed predominantly by commercial bank loans, especially after the market-oriented reform in 2006, when government investment guarantees were abandoned. Commercial capital markets were envisaged to improve efficient capital allocation and management. We analyzed the effects of commercial bank loans on interest rates, investments and allocative efficiency in the Dutch healthcare sector. We aimed to explain variation in interest rates by financial performance of healthcare providers, hypothesizing that the reform reduced interest rates for financially well-performing providers. Using financial data from publicly available annual reports, we explored the effect of financial performance on long-term loan interest rates through pooled linear regressions. Our data showed that financial reserves have steadily increased, although profitability margins have declined since 2011–2013 (depending on the sector). While nominal interest rates have generally declined since 2006, the risk surplus on healthcare loans has steadily increased. Furthermore, we observed no significant relation between the financial performance of healthcare providers and interest rates on capital loans. Maintaining additional financial reserves provided no apparent benefit to capital costs. This suggests that healthcare providers may consider whether financial reserves should be maintained at current levels or can better be used for direct investments. Moreover, healthcare policymakers should evaluate whether the increase in risk surplus combined with an apparent lack of reward for financial scrutiny is a desired outcome of the reform.

**Supplementary Information:**

The online version contains supplementary material available at 10.1007/s10198-023-01629-z.

## Introduction

In market-oriented systems, providers rely on private capital markets to obtain funding. Private lenders may be better than governments in stimulating efficient use of funds, i.e., fund the most promising healthcare investment opportunities [1]. Although most healthcare systems still rely mostly on government funding to finance large-scale investments, some systems display a more prominent role for private capital markets [2].

In this respect, the Netherlands is an interesting case. Market-oriented reforms gave a prominent role to the private capital markets, which in combination with bans on profit distribution, resulted in an almost full reliance on banks to finance investments [3, 4]. In 2006, the Dutch government chose to reform the healthcare system based on Enthoven’s model for managed competition [5]. It was presented as a platform to increase efficiency, guard solidarity and provide freedom of choice, accessibility and financial sustainability [3–6].

A key part of the reform was aimed at providing the healthcare sector with more freedom to invest as it preferred. This was achieved by the termination of the Healthcare Planning Act (HPA; in Dutch: *Wet *“*ziekenhuisvoorzieningen*”). The HPA obliged healthcare providers to apply for a governmental certificate, which was necessary for major construction for healthcare facilities. This certificate acted as a governmental tool to limit new construction and place budget ceilings on construction projects. This certificate also allowed healthcare providers to receive full reimbursement for depreciation and other capital costs (interest charges). As reimbursement was warranted by the government under the old legislation, the risk assessment by the financial institutions that provided the loans was noticeably low, cost price with a small surplus at most. In addition, the loans were provided with long repayment plans, going up to periods of 45 years [3, 6, 7].

Financial institutions thus gained a more dominant role in assessing healthcare capital investments (e.g., land, construction, and equipment). It was argued that governmental hospital planning would interfere with the proposed competition of the new system [8]. Furthermore, as depreciation and capital costs were basically risk free (covered by the government), it would limit incentives to promote for an efficient use of capital. The Healthcare Admission Act (HAA; in Dutch: “*Wet Toelating Zorginstellingen*”*)* replaced the Healthcare Planning Act in 2006 [9]. Under this act, (new) providers are required to conform to state requirements regarding, for example, access and financial transparency, but they are no longer dependent on state capacity planning for large investments. Additional regulation prohibited profit payouts for large parts of the (inpatient) healthcare sector. This banned providers form tapping private capital on the stock markets, and effectively forced them to be reliant on bank loans—and retained earnings—to fund their investments. Efforts to abolish this prohibition of the distribution of dividends have failed up to now [10, 11].

Starting from 2008, hospital capital costs were incorporated in the Dutch DRG-like payments (in Dutch: *diagnose-behandelcombinatie*), which were, to a large extent, negotiable with healthcare insurance companies [4, 12]. This was implemented incrementally [13]. After temporary safety nets, providers would face full risks on capital costs and depreciation as of 2016 [14, 15]. During this period, governmental warrants for capital costs were gradually lowered. Capital costs were gradually integrated into reimbursements for healthcare services. For the long-term care sector, government planning was abolished in 2009. After temporary safety nets, providers would face full risks as of 2016 as well [8], and the cost of capital was integrated into the long-term care payment scheme [16].

These legislative changes transferred investment risk from the government toward the private banks—and the healthcare providers. Furthermore, the European BASEL III and Solvency 2 legislation limited risky behavior of financial institutions [17]. Following these changes, financial institutions more critically evaluated capital loans for the healthcare sector. In addition, financial institutions installed additional requirements to reduce their risk, such as limits to the loan period or the amount of capital a healthcare provider can borrow. Banks shortened investment periods for new property developments from 45 years to 25 [3] and limited loans to €70 million per lender [17]. This effectively forces healthcare providers to borrow from a consortium of institutions in major property development projects [17].

To prepare for and offset the increased investment risk healthcare providers bore, the government established the Healthcare Sector Guarantee Fund in 1999 *(*HGF; in Dutch:* “Waarborgfonds voor de Zorgsector”*). This national fund provided the healthcare sector with a guarantee on capital loans. It was demonstrated that a guarantee from the HGF can provide a 1% up to a 1.5% benefit on real interest charges over the period 2015–2020 [18]. However, financial criteria to enter the fund include an established financial stability, which is to be reviewed by the HGF. Healthcare providers also contribute a small percentage of their yearly revenue to the HGF in a fund that protects against default cases [18].

The interest rates on loans represent costs that the borrower pays for capital. These interest rates are often expressed as a percentage of the principal amount. How financial institutions set interest rates on loans is affected by current inflation rates and by supply and demand of capital and monetary policy of the government [19–21]. Besides systemic macroeconomic effects, idiosyncratic factors related to individual investment and borrower characteristics can also affect interest rates. Some of these factors are captured in the terms of the loan. Such factors are, but are not limited to, the principal amount, the period of the loan term, the provided guarantee and the method of loan repayment. It is well established that borrowers have to pay more for riskier investments [22].

The aim of this study was to gain an understanding on how the capital market liberalization in the Dutch healthcare sector has affected the risk surcharge on long-term bank loans and financial performance of healthcare providers. We expected that this reform increased the efficiency of capital allocation (i.e., allocation of capital to sensible investment plans and lower interest rates for financially stable organizations). We hypothesized an increase in interest rate spread, which reflects investment risks for commercial banks. We also expected total investments to keep growing in line with total healthcare expenses, to upkeep infrastructure with growing service provision. In response to the more strict requirements from capital providers, we hypothesized that healthcare providers have substantially bolstered their financial position [3, 23]. Economic theory presumes that a stronger financial position would result in lower investment risks and subsequently result in lower interest rates. Thus, we were able to test whether commercial financing by banks is efficient. We (1) mapped the changes in interest rate spread, (2) showed trends in capital investments, (3) outlined any changes in financial positions of healthcare providers and (4) explored whether a stronger financial position results in a lower interest rate on capital loans.

## Methods

### Research design

We assessed whether providers with good financial performance obtain relative lower interest rates on their loans. This would stimulate providers to improve financial performance. Analysis consisted of two steps: (1) trends in interest rates per sector, total capital investments per sector and financial performance per sector; (2) conducting a pooled regression analysis to investigate the relation between the financial performance and interest rates for individual healthcare providers.

### Data collection and transformation

The data used in this study were collected from the DigiMV database, a publicly available collection of annual financial reports of Dutch healthcare providers [24]. Publishing annual reports is mandatory for each provider under the Healthcare Admission Act (HAA) (*Wet Toelating Zorginstellingen*). The healthcare providers are obliged to upload their data in unified formats and in certified annual reports. Small providers (under 10 employees) are exempted. This data covers over 95% of providers in medical and long-term care. Included sectors are general hospitals, university medical centers, nursing homes and home care, mental healthcare, disability care, independent treatment centers and revalidation care. Continuous variable outliers of financial ratios selected through analysis (1.5 interquartile range below first and above the third quartile) were inspected on correctness and transformed where applicable. Transformation occurred based on the variable value presented in the annual financial report. String-based variables were unified by a unification key and manually checked to correct for spelling (e.g., abbreviations and capital use). Data were analyzed using RStudio (version 4.2.0) [25].

### Analysis

We plotted median provider interest rates over time per sector and compare these to the Dutch 10-year government bond interest rate. This demonstrated the opportunity costs of loans. Loans provided to the Dutch government are perceived as relatively risk free, represented by a triple A rating [26]. We defined the interest charges added to this risk-free interest rate as the risk surplus, which is related to the perceived risk of the capital investment by the lender, i.e., the risk of lender bankruptcy relative to the Dutch government [27]. Additionally, we separated loans provided by the HGF, as this was expected to result in lower interest rates.

Second, we plotted total capital investments and financial performance of healthcare providers who obtained capital loans over time. Total capital investments include short-term investments in capital goods, such as computers and diagnostic devices, as well as long-term investments in buildings and facilities. Financial performance was plotted for each indicator separately as well as for a composite score of all indicators. To construct a composite score, we transformed financial ratios to a standard-normal distribution (*Z*-score transformation) and calculated the mean *z*-score per provider, reflecting a financial benchmark score (positive if above average, negative if below average).

**Fig. 1:**
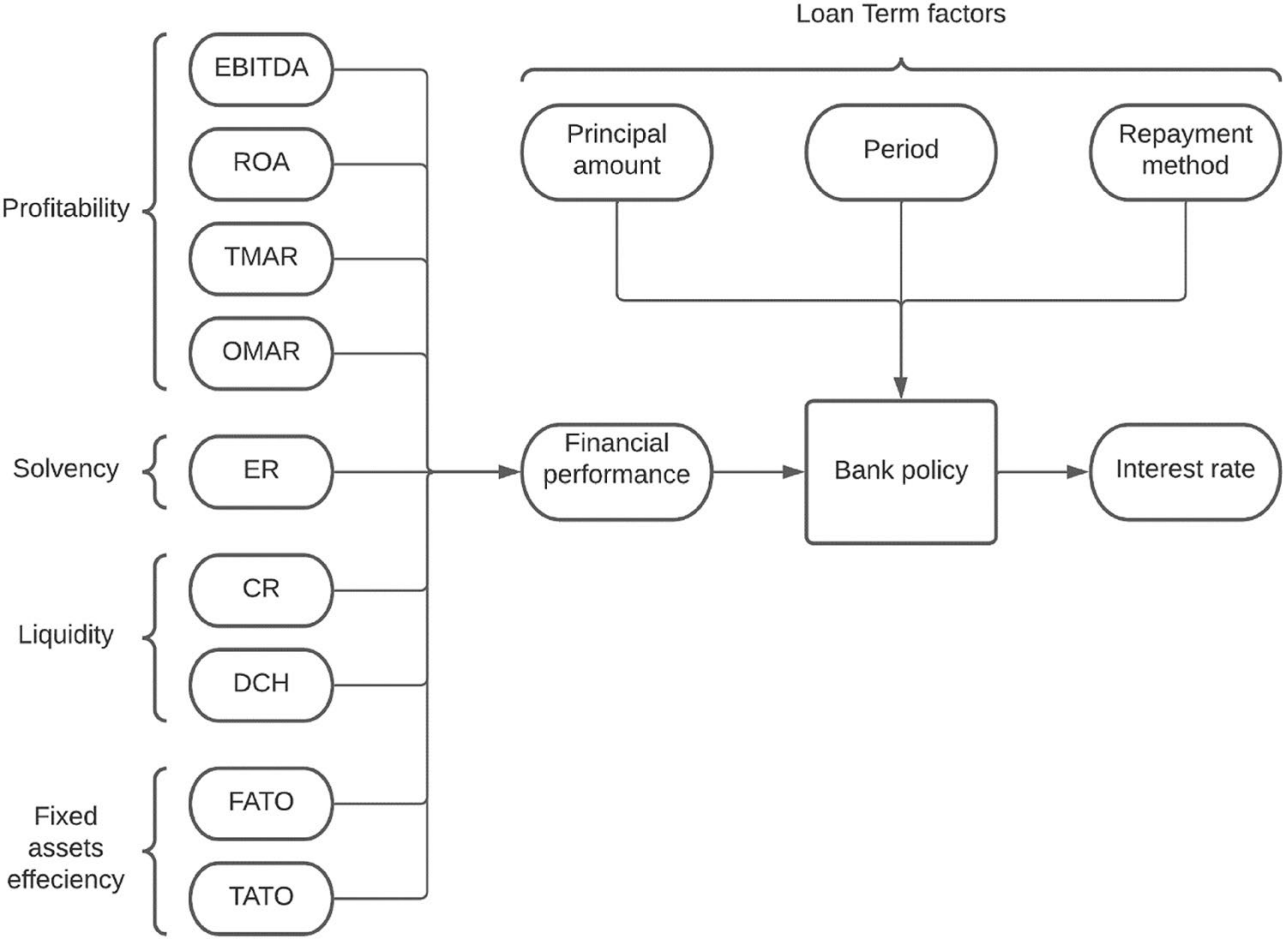
Conceptual framework research project. *EBITDA *earnings before interest, taxes, depreciation and amortization; *ROA* return on total assets; *TMAR* total margin; *OMAR* operating margin; *ER *equity ratio; *CR* current ratio; *DCH *days cash on hand; *FATO* fixed assets turnover; *TATO *total assets turnover

We utilized a subset of the ratios commonly used by Dutch banks to measure the financial provider performance. This set of ratios was based on a factor analysis performed by Zeller et al. [28], amended by Dutch literature (See Table 1) [18, 29]. To assess profitability characteristics, we used the ratios ROA, TMAR, OMAR and EBITDA. We used the solvency ratio ‘equity ratio’ (ER) as an indicator for financial reserves in the organization. This is probably the most important criterion for providers to get access to the underwriting by both the Dutch HGF and the commercial banks (minimal ER of 20–25%) [18, 29]. Liquidity is another characteristic which HGF clarifies as important. We included the current ratio (CR) and the ‘days cash on hand’ (DCH). Finally, to assess fixed assets efficiency, we utilized both the fixed assets turnover ratio (FATO) and the total assets turnover ratio (TATO). These ratios show how efficiently the organization can generate revenue from their assets. Due to lack of data availability, we excluded the characteristics ‘fixed asset age’ and ‘debt coverage’ from our framework. Moreover, ‘debt coverage’ brought inherent statistical problems, as our aim was to investigate the correlation with the interest rates. We adjusted for loan characteristics affecting the interest rate beyond the financial performance of the healthcare provider, such as the principal amount, the loan period and the method of repayment, as shown in Fig. 1.

**Table 1:** Financial characteristics and corresponding ratios relevant to the healthcare sector (from Zeller et al. [28])

Financial characteristic	Ratio	Definition	References
Profitability	Return on total assets (ROA)	Revenues and gains in excess of expenses and losses/total assets	[28, 30–34]
	EBITDA	Earnings before interest, taxes, depreciation and amortization	[18, 29]
	Total margin (TMAR)	Revenues and gains in excess of expenses and losses/total revenue + net nonoperating gains	[28, 31, 34]
	Operating margin (OMAR)	Total revenue − total expenses/total revenue + net nonoperating gains	[28, 34–36]
	Revenue per patient (RPP)	Operating revenue/discharge	[30, 34, 36]
	Operating margin-PLA (OMRPL)	Total revenue − total expenses + depreciation − price-level depreciation/total revenue + net nonoperating gains	[28]
Fixed asset efficiency	Fixed asset turnover (FATO)	Total revenue + net nonoperating gains/net fixed assets	[28]
	Fixed asset turnover-PLA (FATOPL)	Total revenue + net nonoperating gains/price level adjusted net fixed assets	[28]
	Total asset turnover (TATO)	Total revenue + net nonoperating gains/total assets	[28]
Capital structure	Equity ratio (ER)	Fund balance/total assets	[28]
	Fixed asset financing (FAF)	Long-term liability/net fixed assets	[28]
Fixed asset age	Average age of physical plant (AAP)	Accumulated depreciation/depreciation expense	[28]
	Depreciation rate (DEPR)	Depreciation expense/gross fixed assets	[28]
Working capital efficiency	Current ratio (CR)	Current assets/current liabilities	[28]
	Current asset turnover (CATO)	Total revenue + net nonoperating gains/current assets	[28]
	Days cash on hand (DCH)	Cash + marketable securities + unrestricted investment/[(total expenses − depreciation)/365]	[28]
Liquidity	Days cash on hand (DCH)	Cash + marketable securities + unrestricted investment/[(total expenses − depreciation)/365]	[28, 37]
	Replacement viability (REPV)	Restricted plant fund balance + unrestricted investments/price-level accumulated adjusted depreciation	[28]
Debt Coverage	Debt service coverage (DSC)	Cash flow + interest expense/principal payment + interest expense	[28]
	Times interest earned (TIE)	Revenues and gains in excess of expenses and losses + interest expense/interest expense	[28]

*PLA* price level adjusted

Figure 1 demonstrates the conceptual approach of this study. In reality, other (qualitative) factors may impact the bank policy regarding interest rates as well, such as assessment of the investment plans and competition with other nearby providers.

We performed a pooled regression analysis to investigate the relation between provider financial performance and the interest rates on loans. A reduced-form regression was performed with the interest rate as the dependent variable and the *Z*-composite score as the independent variable. Additionally, we included time dummies to correct for the year effect. Moreover, we clustered robust standard errors at the provider level to correct for covariance in loans of the same provider. The robust standard errors also correct for heteroskedasticity. Following the reduced-form regression model, we expanded with multiple control variables. These include the principal amount borrowed, loan period, specified healthcare sector, a HGF guarantee and the method of loan repayment. The models are specified below.

Reduced-form model:

$$\begin{aligned} {\text{Interest}}\;{\text{rate}}_{{{\text{Observation}}\;{\text{Provider}}\;{\text{(op)}}}} = & \beta_{0} + \delta_{0} \cdot {\text{year}}\;{\text{dummy}} + \ldots + \delta_{0(n)} \cdot {\text{year}}\;{\text{dummy}}_{(n)} \\ \quad + \beta_{1} \cdot {\text{financial}}\;{\text{performance}}_{{{\text{1op}}}} + u_{{\text{o}}} + u_{{\text{p}}} . \\ \end{aligned}$$

Expanded model:

$$\begin{aligned} {\text{Interest}}\;{\text{rate}}_{{{\text{Observation}}\;{\text{Provider}}\;{\text{(op)}}}} = & \beta_{0} + \delta_{0} \cdot {\text{year}}\;{\text{dummy}} + \ldots + \delta_{0(n)} \cdot {\text{year}}\;{\text{dummy}}_{(n)} \\ \quad + \delta_{0} \cdot {\text{control}}\;{\text{dummy}} + \ldots + \delta_{0(n)} \cdot {\text{control}}\;{\text{dummy}}_{(n)} \\ \quad + \beta_{1} \cdot {\text{financial}}\;{\text{performance}}_{{{\text{1op}}}} + \beta_{2} \cdot {\text{ControlVar}}_{{{\text{1op}}}} + u_{{\text{o}}} + u_{{\text{p}}} . \\ \end{aligned}$$

## Results

### Increased risk surplus on loans

**Fig. 2:**
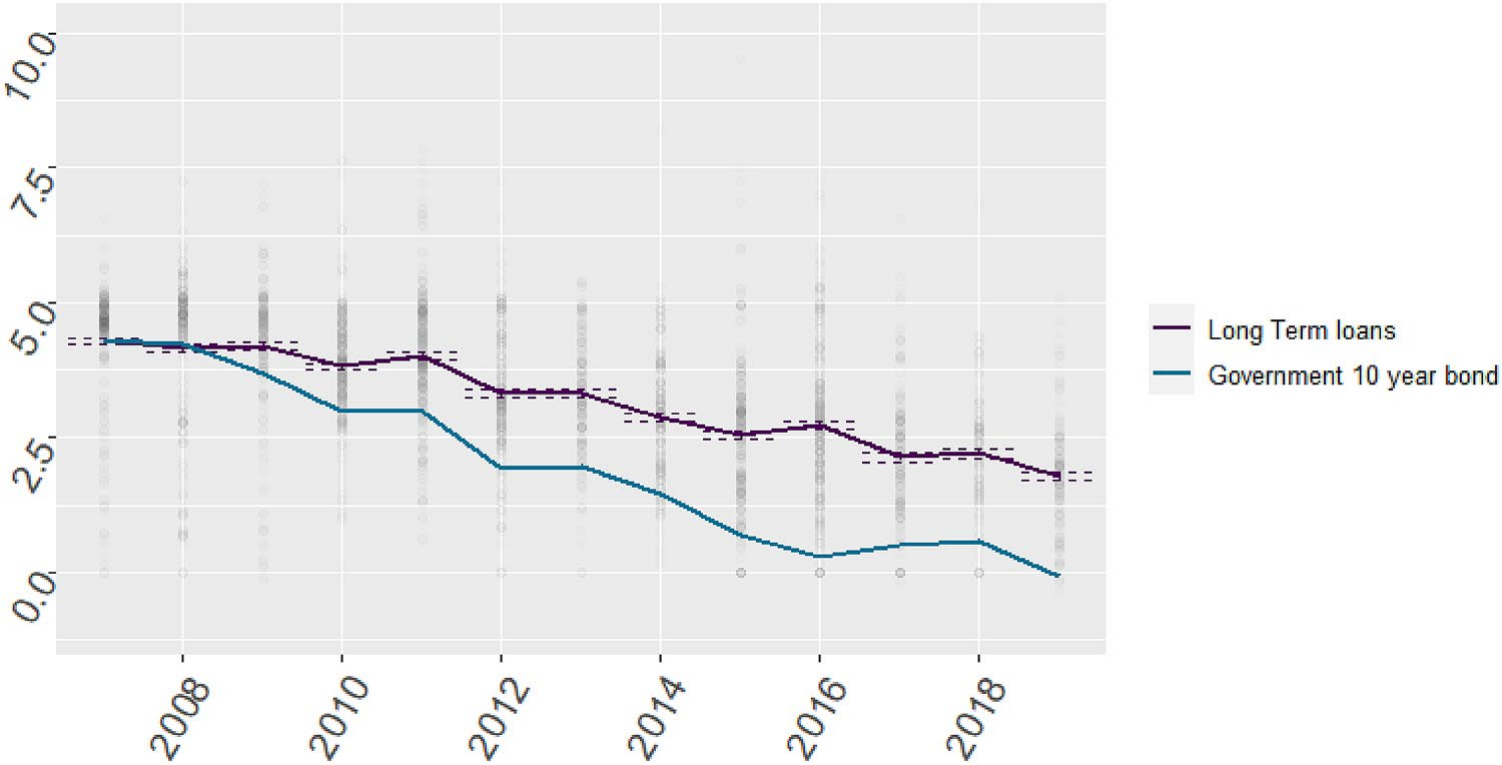
Time series of interest rates for long-term banking loans in the healthcare sector compared to interest rates (%) for government bonds. Dots represent individual observations (loans)

We mapped the changes in interest rates for the entire healthcare sector (including university medical centers, general hospitals, disability care, mental healthcare, revalidation care and independent treatment centers) in Fig. 2. We clearly demonstrated a declining trend in interest rates, from 4.5% in 2007 to 2.5% in 2019. However, the interest rates on 10-year government bonds showed even larger declines of 4.5% in 2007 to 0.0% in 2019. The interest rate spread between long-term loans and government bonds has thus increased since 2007. This could signal for example that commercial banks rate healthcare loans as increasingly riskier, or that banks receive additional markups on healthcare loans unrelated to the level of risk, such as growing market power of lack of competition.

### Declining investments

**Fig. 3:**
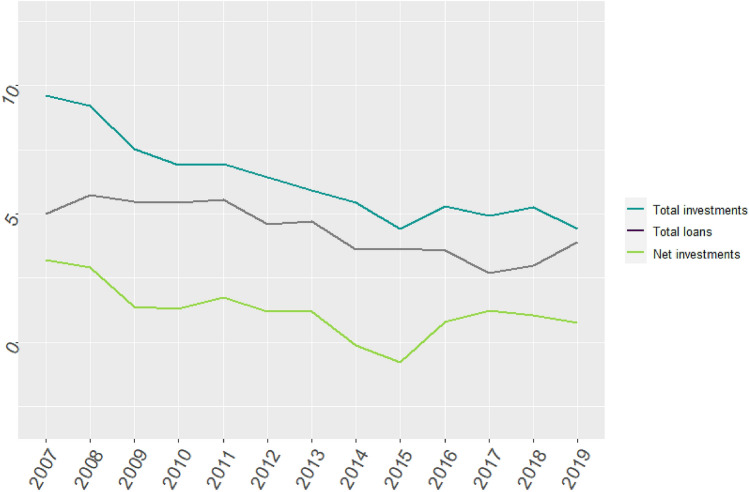
Decline in investments in the Dutch healthcare sector (in % of revenue). Net investments represent mutations in fixed assets (i.e., total investments after depreciation)

Investments have declined over the period 2007–2019 (Fig. 3). In 2007, approximately 10% of total revenue was used for investments, compared to only 5% in 2019. About half of the investments in 2007 were financed through capital loans, and this has increased slightly from 2008 onward. This may indicate that retained earnings were used less for investments, and more so for improving the financial position (ER). Net investments were indicated by mutations in fixed assets, which express the value of total investments after depreciation.

### Improvements in financial position for healthcare providers

**Fig. 4:**
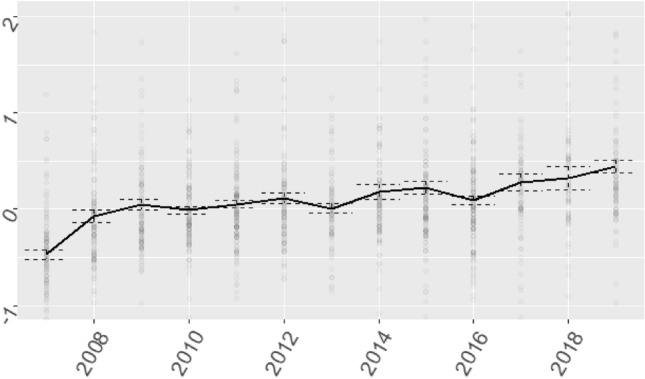
Time series of *Z*-composite score of healthcare providers which procured a capital loan during the study period (2007–2019). Dots are individual healthcare providers

**Fig. 5:**
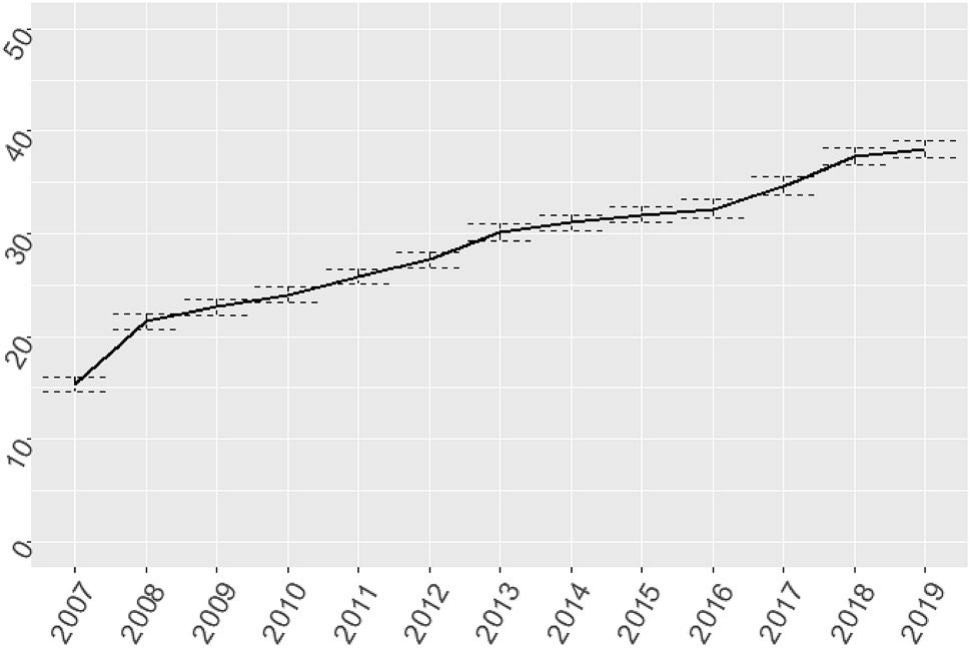
Time series of equity ratio (ER, %) of healthcare providers which procured a capital loan during the study period (2007–2019)

Figure 4 shows the financial *Z*-composite score. The *Z*-composite score shows the relative financial performance of an organization compared to the average of the entire sector. ROA, EBITDA margin, ER, CR, DCH, FATO and TATO are included in this score. Financial positions increased across the board over the period 2007–2019, although significant variation remains (Fig. 4). A separate trend analysis of the ER iss included in Fig. 5, which demonstrates that financial reserves have doubled over the study period. This is in line with trends in investments in the healthcare sector (Fig. 3), as investments are increasingly financed through capital loans, rather than financial reserves.

### No relation between financial position and interest rates

While overall trends showed a discrepancy between both higher surpluses on interest rates on the one hand and an increased financial performance on the other hand, this could be due to composite effects (e.g., ecological fallacies) or confounders. We tested whether an association exists at the provider level, i.e., whether good financial performance pays off in terms of lower relative interest rates. This was tested by pooled linear regression, as shown in Table 2. These results demonstrated that financial performance of healthcare providers had no significant relation with the interest rates on long-term banking loans, neither in the reduced-form model or in the expanded model. Robustness checks found either no correlation or significant correlation with wrong signs for individual indicators (Supplementary material 1). For example, a higher EBITDA was associated with higher interest rates.

**Table 2:** Regression analysis estimating the effect of financial performance (*Z*-composite) on interest rates

	Reduced-form regression			Extended regression		
	Estimate	SE	*P* value	Estimate	SE	*P* value
*Z*-composite	0.055	0.036	0.129	0.064	0.034	0.057
Principal amount (Ln)	0.073	0.022	0.001*	0.061	0.024	0.013*
Loan period (Ln)	0.256	0.055	0.000*	0.299	0.061	0.000*
Revenue (Ln)	−0.087	0.017	0.000*	−0.072	0.017	0.000*
HGF guarantee	−0.639	0.044	0.000*	−0.537	0.053	0.000*
Sector (reference = UMC)						
General hospital	0.175	0.063	0.006*	0.209	0.063	0.001*
ITC	0.260	0.072	0.000*	0.188	0.075	0.012*
Elderly care	0.010	0.053	0.855	0.014	0.053	0.795
Disability care	−0.062	0.057	0.275	−0.077	0.057	0.179
Mental healthcare	0.117	0.063	0.063	0.064	0.063	0.311
Revalidation care	−0.237	0.118	0.045*	−0.276	0.114	0.016*
Bank (reference = bank 1)						
Bank 2				−0.129	0.173	0.457
Bank 3				−0.642	0.086	0.000*
Bank 4				0.183	0.180	0.309
Bank 5				−0.164	0.231	0.477
Bank 6				−0.689	0.110	0.000*
Bank 7				−0.081	0.240	0.736
Bank 8				0.332	0.426	0.435
Bank 9				0.040	0.229	0.861
Bank 10 (international)				−1.448	0.291	0.000*
Bank 11				−0.328	0.088	0.000*
Bank 12				0.438	0.509	0.390
Bank 13				−0.952	0.129	0.000*
Bank 14				−0.454	0.100	0.000*
Bank 15				−0.597	0.089	0.000*
Bank 16				0.248	0.622	0.691
Bank 17				−0.500	0.134	0.000*
Repayment method (reference = interest free)						
Annuity				0.203	0.234	0.386
Balloon				0.644	0.211	0.002*
Bullet				0.160	0.295	0.587
Combination				0.117	0.337	0.729
Linear				0.052	0.175	0.766
Other				0.338	0.323	0.295
Predefined scheme				0.420	0.547	0.443
Variable				0.479	0.343	0.163
Fixed amount per period				0.413	0.190	0.030*
Year (reference = 2007)						
2008	−0.219	0.106	0.038*	−0.207	0.109	0.059
2009	−0.104	0.106	0.325	−0.008	0.106	0.941
2010	−0.522	0.083	0.000*	−0.464	0.086	0.000*
2011	−0.341	0.088	0.000*	−0.276	0.088	0.002*
2012	−1.038	0.095	0.000*	−0.945	0.098	0.000*
2013	−1.028	0.098	0.000*	−0.961	0.101	0.000*
2014	−1.433	0.097	0.000*	−1.365	0.102	0.000*
2015	−1.866	0.099	0.000*	−1.801	0.103	0.000*
2016	−1.649	0.096	0.000*	−1.602	0.099	0.000*
2017	−2.256	0.105	0.000*	−2.163	0.107	0.000*
2018	−2.256	0.110	0.000*	−2.142	0.117	0.000*
2019	−2.612	0.106	0.000*	−2.543	0.112	0.000*
Intercept	4.253	0.375	0.000*	4.344	0.453	0.000*
*N*	3615			3408		
Adjusted *R*^2^	0.351			0.384		

Analysis performed with the basic and extended model containing a pooled regression analysis with robust and clustered standard deviation at provider level. Values marked with * are significant at *P *< 0.05

*SE *standard error; *UMC *University Medical Center; *ITC *independent treatment center; *HGF *healthcare guarantee fund

The results for the expanded model show that the duration of the loan had a positive association with interest rates, suggesting that an increase in loan period duration can result in higher interest rates. The size of the healthcare organization in terms of revenue is associated with lower interest rates. This is confirmed by the relatively low interest rates that university medical centers paid, compared to general hospitals and other providers. Moreover, the results indicated that a HGF guarantee yielded a significant discount on the interest rate over the studied time period. Finally, we observed significant differences between individual banking organizations. A foreign bank had significantly lower interest rates as compared to national banking organizations.

Sensitivity analyses found a negative association between financial performance and the principal amount of the loan, disproving the hypothesis that financial position offers benefits through higher principal amounts (Supplementary material 2). A lag on the *Z*-composite score, which assumes that banks have a delayed response to financial performance, did not result in a significant effect on interest rates, similar to our main analysis (Supplementary material 3).

## Discussion

We analyzed the interest rates on long-term banking loans of capital investments and their relation with the financial performance of healthcare providers in the Dutch healthcare system. We found financial positions did improve and so did the calculated surplus on the risk-free healthcare interest rates. However, we found no evidence that interest rates on individual loans were associated with financial position, questioning the premise that improved financial scrutiny by providers pays off in terms of lower interest rates. This suggests that these capital market reforms brought additional costs to the health system (higher real interest rates) without providing clear incentives for providers to improve their financial position, which questions whether a liberalized capital market for healthcare providers results in efficient capital allocation.

Our unexpected results may be due to the fact that general interest rate trends in the healthcare system correspond to increased risk perception by banks. For example, financial institutions have lowered their loan terms from 45 to 25 years in response to the capital market reforms [3, 22]. Basel III has been implemented in 2011, which incentivizes banks to put a higher price tag on investment risk. This could possibly explain the increase in risk surplus on the interest rates we observed from 2011 and onward [38, 39]. Furthermore, the healthcare sector saw a few major bankruptcies since the reform^1^ and these can have major impact on views of the sector’s stability and may increase risk perception [40]. However, all of these trends apply to the sector as a whole, not necessarily to individual providers. Also, large hospitals (i.e., university medical centers) that politically are too big to fail did not get a large discount. Furthermore, a significant percentage of loans is safeguarded against bankruptcy by the HGF [18]. While this was indeed associated with average rate reductions of 0.5%, significant positive markups still remain. Our analysis of the HGF impact included a longer period compared to the previous evaluation (2007–2019 and 2015–2020, respectively) and other confounders may have been used in analysis [18]. To sum up, we found no clear evidence that financial performance and interest rates correlate on the provider level and this suggests that increased markups are not fully reflective of increased risk perception.

Healthcare loans are relatively profitable for banks. Given that in the Netherlands a few major banks provide the bulk of health capital, our results could also be explained by a lack of competition in the capital markets for provider loans. Since healthcare providers have few options but to rely on a small number of national banks for capital, the market is prone to oligopolistic competition and excess prices. This is supported by anecdotal evidence of some hospitals acquiring capital from international investors at low interest rates [41]. Concerns were raised about the decline in investments in healthcare [3]. This trend is also consistent with bank oligopoly behavior, where capital supply is constraint to (further) boost interest rates. From the provider perspective, investments through long-term bank loans carry more risk compared to before the reform, which results in more scrutiny toward investments. This may not necessarily be inefficient, as in absence of financial risk providers may have had incentives to maximize investments before the reform. A short-term decline in investments may have been expected as a result of the reform, once banks induced scrutiny in investment decisions. Underinvestment in the healthcare sector is currently a real risk, given that capital loans have become more expensive over time and healthcare providers are encouraged to increase financial reserves, while major investments are required (e.g., digitalization and sustainability).

### Limitations and future research

This study is the first that aimed to relate commercial healthcare interest rates to provider financial position over longer time periods, rendering evidence on private capital market performance to fund a fully non-profit healthcare provider sector. Our research provided strong evidence that the capital market reform did not live up to its expectations in improving financial performance of healthcare providers. However, a few limitations should be noted. First, our selection of financial ratios was based on the analysis of Zeller et al., supplemented with commonly used ratios in the Dutch healthcare sector. Although extensive, these ratios might not fully capture the financial performance of healthcare providers. Second, we only observed loans that were realized, introducing selection bias toward successful bargaining outcomes. If providers with low financial stability are unable to obtain loans, market discipline would be present but would not be detected in our current research design. Third, banks might outperform the government in evaluation of healthcare business plans, irrespective of financial position. This would induce efficiency in capital use in healthcare which is not included in our design. However, the decline in healthcare investments can be attributed to a more stringent evaluation process of capital loans by banks. Fourth, competition among providers was not taken into account, which may influence interest rates. For example, hospitals with few competitors nearby may have a higher bargaining position irrespective of financial position. Finally, the publicly available dataset used in this study is self-reported by healthcare organizations. Corrections were applied to mitigate input errors, but some small errors may remain. Given the large set of observations, however, the risk of distorting the analysis is relatively low.

### Policy implications

In the Netherlands, healthcare capital is predominantly financed through bank loans. This is potentially an interesting financing model for countries that predominantly rely on government financing. The Dutch case reveals that, under certain conditions, a bank finance reform may reduce investments in healthcare, and at the same time may increase the cost of capital. While loan warrants, such as those provided by the HGF, may reduce the cost of capital, they may be insufficient to reward providers for financial scrutiny. Increased competition in the banking sector may reduce the cost of capital while increasing the incentives for providers to increase their financial position. Governments may also allow for alternative sources of capital (e.g., equity capital) to increase competition. Some studies hint that private equity may increase total health expenses [42], although evidence is mixed [43].

The predominant position of three Dutch banks in healthcare financing begs the question to what extent these banks affect healthcare policy and planning. For example, banks may have a major role in hospital bankruptcy decisions, mergers, or primary care substitution policies. Additional research is needed to assess the role of banks as stakeholder in policy formation.

## Conclusion

This study showed that interest rate markups for healthcare providers have increased in the years since the market-oriented reform of the Dutch healthcare system. Simultaneously, healthcare providers improved their financial position, especially through extensive buildup of financial reserves. Counterintuitively, we found no significant relation between the financial performance of providers and what they pay on the interest rate of long-term banking loans. Policy makers should question whether these contribute to a financially sustainable healthcare system, especially if additional sources of investment are needed due to aging and the digitalization of the health sector. Healthcare providers may consider whether they continue to increasing their financial reserves to obtain benefits in capital costs for future investments, or if they are better off by investing these reserves directly.

### Supplementary Information

Below is the link to the electronic supplementary material.

Supplementary file1 (DOCX 15 KB)

Supplementary file2 (DOCX 16 KB)

Supplementary file3 (DOCX 16 KB)

## Footnotes

1 Most notably long-term care provider Meavita (2009), the ‘Ruwaard van Putten Hospital’ (2013), Sionsberg Hospital (2014), and the Slotervaart Hospital and IJsselmeer Hospitals (2018).
