# Supplementary material for: Assessing the relation between financial performance and long-term bank loan interest rates for healthcare providers in the Netherlands: a panel data analysis
Source: Eur J Health Econ. 2023 Sep 13;25(5):845–55. doi: 10.1007/s10198-023-01629-z (PMC11192655; doi:10.1007/s10198-023-01629-z)
Supplement: Supplementary file 1 — Supplementary file1 (DOCX 15 KB) [file 10198_2023_1629_MOESM1_ESM.docx]

Assessing the relation between financial performance and long-term bank loan interest rates for healthcare providers in the Netherlands: a panel data analysis

**Journal**

European Journal of Health Economics

**Author information**

1. Erik Wackers, MSc (corresponding author)^1^

ORCID: 0000-0002-0290-7103

2. Rick Smit, MSc (shared first co-authorship)^1^

ORCID: 0000-0003-0373-8484

3.Niek Stadhouders, PhD^1^

ORCID: 0000-0002-7296-2335

4.Patrick Jeurissen, PhD^1^

ORCID: 0000-0002-4198-2448

^1^Radboud University Medical Center, Radboud Institute for Health Sciences, IQ healthcare, Nijmegen

**Supplementary material 1**. Summary of individual regression results for financial indicators included in Z-composite score. Controls for Year, Principal amount, Loan period, HGF Guarantee, Revenue, and Sector.

|  |  | Estimate | SE | P-value |
| --- | --- | --- | --- | --- |
|  |  |  |  |  |
| Financial indicator |  |  |  |  |
|  | ER | -0,204 | 0,194 | 0,293 |
|  | EBITDA | 0,792 | 0,199 | 0,000* |
|  | ROA | 0,815 | 0,513 | 0,122 |
|  | CR | -0,020 | -0,839 | 0,402 |
|  | FATO | 0,001 | 0,001 | 0,0496* |
|  | TATO | -0,017 | 0,047 | 0,719 |
|  | DCH | -0,001 | 0,0003 | 0,051 |
|  |  |  |  |  |

SE = Standard error; EBITDA = Earnings before interest, taxes, depreciation, and amortization; ROA = Return on total assets; ER = Equity ratio; CR = Current ratio; DCH = Days cash on hand; FATO = Fixed assets turn-over; TATO = Total assets turn-over; HGF = Healthcare guarantee fund.
