# Supplementary material for: Assessing the relation between financial performance and long-term bank loan interest rates for healthcare providers in the Netherlands: a panel data analysis
Source: Eur J Health Econ. 2023 Sep 13;25(5):845–55. doi: 10.1007/s10198-023-01629-z (PMC11192655; doi:10.1007/s10198-023-01629-z)
Supplement: Supplementary file 2 — Supplementary file2 (DOCX 16 KB) [file 10198_2023_1629_MOESM2_ESM.docx]

Assessing the relation between financial performance and long-term bank loan interest rates for healthcare providers in the Netherlands: a panel data analysis

**Journal**

European Journal of Health Economics

**Author information**

1. Erik Wackers, MSc (corresponding author)^1^

ORCID: 0000-0002-0290-7103

2. Rick Smit, MSc (shared first co-authorship)^1^

ORCID: 0000-0003-0373-8484

3.Niek Stadhouders, PhD^1^

ORCID: 0000-0002-7296-2335

4.Patrick Jeurissen, PhD^1^

ORCID: 0000-0002-4198-2448

^1^Radboud University Medical Center, Radboud Institute for Health Sciences, IQ healthcare, Nijmegen

**Supplementary material 2.** Regression analysis with principal amount as dependent variable. Values marked with * are significant at P < 0.05.

|  |  | Reduced-form regression | | |
| --- | --- | --- | --- | --- |
|  |  | Estimate | SE | P-value |
| Z-composite |  | -0,313 | 0,032 | 0,000* |
| Year (Reference = 2007) |  |  |  |  |
|  | 2008 | 0,073 | 0,076 | 0,340 |
|  | 2009 | -0,039 | 0,085 | 0,648 |
|  | 2010 | 0,243 | 0,067 | 0,000* |
|  | 2011 | 0,339 | 0,068 | 0,000* |
|  | 2012 | 0,265 | 0,073 | 0,000* |
|  | 2013 | 0,392 | 0,076 | 0,000* |
|  | 2014 | 0,196 | 0,081 | 0,015* |
|  | 2015 | 0,292 | 0,083 | 0,000* |
|  | 2016 | -0,045 | 0,076 | 0,554 |
|  | 2017 | 0,186 | 0,095 | 0,051 |
|  | 2018 | 0,219 | 0,099 | 0,027* |
|  | 2019 | -0,029 | 0,112 | 0,798 |
| Loan period (Logarithmic) |  | 0,440 | 0,046 | 0,000* |
| HGF Guarantee (Reference = 0; no guarantee) |  | 0,010 | 0,036 | 0,774 |
| Sector (Reference = University medical center) |  |  |  |  |
|  | General hospital | 0,161 | 0,054 | 0,003* |
|  | Independent treatment center | -0,142 | 0,067 | 0,033* |
|  | Nursing homes and home care | -0,145 | 0,044 | 0,001* |
|  | Disability care | -0,321 | 0,052 | 0,000* |
|  | Mental healthcare | -0,123 | 0,054 | 0,024* |
|  | Revalidation care | 0,118 | 0,086 | 0,171 |
| Revenue (Logarithmic) |  | 0,378 | 0,016 | 0,000* |
| Interest rate |  | 0,048 | 0,015 | 0,001* |

SE = Standard error; HGF = Healthcare guarantee fund
