# Supplementary material for: Assessing the relation between financial performance and long-term bank loan interest rates for healthcare providers in the Netherlands: a panel data analysis
Source: Eur J Health Econ. 2023 Sep 13;25(5):845–55. doi: 10.1007/s10198-023-01629-z (PMC11192655; doi:10.1007/s10198-023-01629-z)
Supplement: Supplementary file 3 — Supplementary file3 (DOCX 16 KB) [file 10198_2023_1629_MOESM3_ESM.docx]

Assessing the relation between financial performance and long-term bank loan interest rates for healthcare providers in the Netherlands: a panel data analysis

**Journal**

European Journal of Health Economics

**Author information**

1. Erik Wackers, MSc (corresponding author)^1^

ORCID: 0000-0002-0290-7103

2. Rick Smit, MSc (shared first co-authorship)^1^

ORCID: 0000-0003-0373-8484

3.Niek Stadhouders, PhD^1^

ORCID: 0000-0002-7296-2335

4.Patrick Jeurissen, PhD^1^

ORCID: 0000-0002-4198-2448

^1^Radboud University Medical Center, Radboud Institute for Health Sciences, IQ healthcare, Nijmegen

**Supplementary material 3.** Regression analysis with lag on z-composite score. Values marked with * are significant at P < 0.05.

|  |  | Reduced-form regression | | |
| --- | --- | --- | --- | --- |
|  |  | Estimate | SE | P-value |
| Z-composite |  | 0,312 | 0,178 | 0,081 |
| Year (Reference = 2008) |  |  |  |  |
|  | 2009 | 0,063 | 0,120 | 0,599 |
|  | 2010 | -0,355 | 0,098 | 0,000* |
|  | 2011 | -0,213 | 0,101 | 0,035* |
|  | 2012 | -0,895 | 0,107 | 0,000* |
|  | 2013 | -0,909 | 0,110 | 0,000* |
|  | 2014 | -1,479 | 0,117 | 0,000* |
|  | 2015 | -1,800 | 0,111 | 0,000* |
|  | 2016 | -1,496 | 0,110 | 0,000* |
|  | 2017 | -2,033 | 0,127 | 0,000* |
|  | 2018 | -2,031 | 0,120 | 0,000* |
|  | 2019 | -2,496 | 0,125 | 0,000* |
| Principal amount (Logarithmic) |  | 0,115 | 0,024 | 0,000* |
| Loan period (Logarithmic) |  | 0,258 | 0,065 | 0,000* |
| Revenue (Logarithmic) |  | -0,082 | 0,020 | 0,000* |
| HGF Guarantee (Reference = 0; no guarantee) |  | -0,642 | 0,049 | 0,000* |
| Sector (Reference = University medical center) |  |  |  |  |
|  | General hospital | 0,122 | 0,075 | 0,106 |
|  | Independent treatment center | 0,397 | 0,087 | 0,000* |
|  | Nursing homes and home care | 0,029 | 0,064 | 0,651 |
|  | Disability care | 0,006 | 0,064 | 0,921 |
|  | Mental healthcare | 0,166 | 0,069 | 0,016* |
|  | Revalidation care | -0,328 | 0,134 | 0,015* |
